# Case Reports of Alopecia Areata Associated With Apical Periodontitis

**DOI:** 10.1155/crid/9446964

**Published:** 2026-09-30

**Authors:** Aleksandra Zuza, Aylin Baysan, Katarina Grcic, Ivona Bago, Hannah Bennett, Flavio Pisani, Shalini Kanagasingam

**Affiliations:** ^1^ Faculty of Medicine, School of Dentistry, Foca University, East Sarajevo, Bosnia and Herzegovina; ^2^ Institute of Dentistry, Faculty of Medicine and Dentistry, Queen Mary University, London, UK, qmul.ac.uk; ^3^ School of Dental Medicine, Department of Endodontics and Restorative Dentistry, University of Zagreb, Zagreb, Croatia, unizg.hr; ^4^ Leeds Teaching Hospitals NHS Trust, Leeds, UK, nhs.uk; ^5^ School of Medicine and Dentistry, University of Lancashire, Preston, UK

**Keywords:** alopecia areata, apical periodontitis, autoimmune disease, endodontic infection, hair loss, root canal treatment, systemic inflammation

## Abstract

Apical periodontitis is an inflammatory response of periradicular tissues caused by microbial infection within the root canal system. Alopecia areata (AA) is a T‐cell mediated, autoimmune condition affecting hair follicles causing nonscarring hair loss, clinically characterised by small patches of baldness on the scalp and/or around the body. Although there have been links to genetic, environmental and hormonal triggers, this condition has rarely been associated with oral infection. This report presents three cases of AA with no obvious aetiology in fit and healthy patients, whereby the alopecic patches appeared coincident with apical periodontitis. The first case describes complete resolution of AA after successful non‐surgical retreatment of the LR6 with apical periodontitis after 3 years. The second case was followed up for 9 months, with a complete healing of AA following the affected tooth extraction, whereas the third case demonstrates complete hair regrowth after 4 months following the single‐visit endodontic therapy. This article highlights the impact of oral infection on endogenic immune‐inflammatory mediators, although causality cannot be inferred from these observations alone. There is a need for an interdisciplinary approach in cases of idiopathic AA, in the absence of other aetiological factors, to preclude unnecessary pharmacological interventions. Due to these limitations, more epidemiological studies would be beneficial to investigate any possible association between AA and apical periodontitis.

## 1. Introduction

Growing evidence suggests that chronic oral inflammatory diseases may exert systemic effects through persistent immune activation and dissemination of inflammatory mediators. Recent studies have highlighted the complex interplay between oral inflammation, host immune responses, microbial burden and environmental risk factors such as smoking, which may contribute to both local tissue destruction and broader systemic inflammatory consequences. Successful management of oral inflammatory conditions has been associated with improvements in biomarkers related to endothelial function, vascular health and systemic inflammatory regulation. These findings support the concept that chronic oral infections may represent a source of sustained immune stimulation capable of influencing extraoral inflammatory and immune‐mediated diseases. Although direct evidence linking apical periodontitis (AP) and alopecia areata (AA) remains limited, the shared involvement of pro‐inflammatory cytokines, T‐cell dysregulation and immune‐mediated tissue responses provide biological plausibility for a potential association and warrants further investigation.

AA is a chronic inflammatory disease that involves hair follicle. Current evidence indicates that hair follicle inflammation in AA is caused by a T‐cell–mediated autoimmune mechanism occurring in genetically predisposed individuals [1]. The associations between AA and atopy, autoimmune thyroid disease and vitiligo were commonly reported. Interestingly, AA affects males and females equally and nearly 2% of the general population experiences at some point during their lifetime. The highest prevalence was reported between the second and fourth decades of life. Up to 66% of patients are below 30 years old, whereas only 20% are older than 40 years old. The most common site to be affected is the scalp. The face, eyebrows, eyelashes, beard, underarm hair and pubic hair may be affected and sometimes, even the entire body. Patches are usually symptom free. However, patients may occasionally describe some tingling, itching or dysesthesia, at times of hair loss [1–3]. Other causes of hair loss which may be present similarly to AA have been compiled in Table 1.

**Table 1:** Differential diagnoses with AP.

Trichotillomania
Temporal triangular alopecia
Central centrifugal cicatricial alopecia
Discoid lupus erythematosus affecting the scalp
Tinea capitis
Telogen effluvium
Androgenic alopecia
Secondary syphilis
Lichen planopilaris
Frontal fibrosing alopecia

The pathogenesis of AA is still unclear. Skin biopsies of the affected skin show a lymphocytic infiltrate hair follicle and genetic studies in patients and mouse models showed that it is complex with polygenic traits [4, 5]. Genome‐wide studies have reported strong associations with immune‐regulation genes such as HLA class II, CTLA4, IL2/IL21 and PTPN22, which encode ligands for the activating receptor NKG2D on cytotoxic T cells [1]. In susceptible patients, the anagen hair follicle which is usually protected from immune attack by low MHC expression and local immunosuppressive signals breaks down. This exposes follicular antigens to the immune system, subsequently activating CD8 + NKG2D + T cells, with support from NK cells and mast cells [1, 3, 6, 7]. Activated the immune cells release IFN‐gamma and IL‐15, which can trigger the JAK‐STAT (Janus kinase‐signal transducer and activator of transcription) signalling pathway which is a central intracellular signalling pathway that drives the expression of chemokines such as CXCL9, CXCL10 and CXCL11, which in turn recruit more T cells to the follicle. The self‐sustaining cycle causes the hair follicle to leave the growth phase (anagen) prematurely and enter regression (catagen), leading to hair loss. Although, the follicle′s stem cells are not destroyed, which explains why hair can regrow once inflammation subsides [6, 8]. Transcriptomic studies of affected skin and blood in AA show a strong type 1 (IFN‐gamma) inflammatory signature, with additional contributions to Th2 and IL‐23 pathways, whereas regulatory T cells (Tregs) are functionally reduced, allowing the autoimmune response to persist. The critical role of these pathways has been demonstrated by the efficacy of JAK inhibitors in reversing disease in both mouse models and patients.

Therefore, the combined therapy involves topical or intralesional corticosteroids, immune therapy with diphenylcyclopropenone and topical immunotherapy [9–11]. There is emerging evidence pointing to a possible association between infected root canal systems and resultant local inflammation leading to destruction of the periradicular tissues, that is, AP and AA. The infectious nidus of AP generating chronic immune stimulation involving pathogen‐associated molecular patterns (PAMPs), cytokines, Th1/Th17 bias and inflammasome activation, may erode hair follicle immune privilege and trigger autoimmunity via cross‐reactivity, bystander activation and systemic pro‐inflammatory signalling, in susceptible individuals. In this respect, eliminating endodontic infectious process could potentially eliminate AA related hair loss. Differential diagnoses of AA are presented in Table 1 [2].

The SALT (Severity of Alopecia Tool) score describes the percentage of scalp hair loss and ranges from 0 to 100. A SALT score of 100 means a complete scalp hair loss. A SALT score of 0 means that there is no scalp hair loss. A SALT score of 50 or greater is generally considered severe disease [12]. Epidemiologic studies have reported that AA is associated with atopic diseases, including asthma, atopic dermatitis and allergic rhinitis. Researchers have implicated autoimmune diseases, particularly thyroid diseases, vitiligo, psoriasis and lupus erythematosus. Patients affected by autoimmune diseases showed a high prevalence of AP, thereby confirming the significant role of an altered immune system in disease pathogenesis [13].

AP is a destructive inflammatory response of periradicular tissues caused by microbial infection within the root canal system. Characterised by persistent inflammatory response and alveolar bone resorption in the periapical region, AP has been shown to affect up to 52% of the global population [14]. The treatment consists of eradicating the microorganisms within the root canal system or substantially reducing the microbial load and preventing re‐infection by root canal filling [15]. When root canal therapy is carried out to an optimum standard, the periapical lesion usually shows sign of healing with periradicular bone regeneration and reduction of the apical radiolucency [16].

Recently, Kim et al. [17] reported that periodontal diseases (PD) may act as a risk factor for AA. However, studies investigating the genetic susceptibilities of PD in AA are lacking evidence. Wang et al. [18] performed a comprehensive bioinformatics analysis to explore common mechanisms of PD and AA. In this respect, AA and derived crosstalk genes enriched biological processes and pathways relevant to PD‐AA linkage highlighted the key diagnostic biomarkers and immune cells that link these two diseases. Interestingly, PD and AA share immune‐related genetic susceptibility loci such as HLA‐DRB1, IL‐1 and IL‐17, along with an inflammatory process and the ratio of Th17 to Treg cells, coupled with overexpression of inflammatory cytokines such as IFN‐*γ* and IL‐17 [19–23]. However, there is limited evidence on the possible association of AA and AP.

The aim of this paper was to present and discuss the manifestations of AA in patients with AP to raise awareness in possible association between AA and AP.

## 2. Case Presentation

Patients were included if they had a dermatologist‐confirmed diagnosis of AA, clinical and radiographic evidence of AP, no known systemic disorders directly associated with hair loss and available follow‐up records. Any cases that exhibited alternative causes of alopecia, incomplete clinical records and the concurrent initiation of systemic immunomodulatory therapy during the follow‐up period were excluded. The key clinical events, diagnostic findings, treatment interventions and follow‐up outcomes are summarised chronologically in Table 2.

**Table 2:** Timeline of diagnosis, intervention and outcomes.

Case	AA history	Dental findings	Intervention	Follow‐up	Outcome
Case 1	Patchy AA at presentation (SALT: 4.8)	AP associated with LR6 and UL6	Retreatment of LR6 followed by extraction of UL6	1 year, 18 months, 3 years	Complete hair regrowth; SALT: 0
Case 2	AA present for 2 years (SALT: 4.0)	AP associated with LL6	Extraction of LL6	3, 6 and 9 months	Complete hair regrowth; SALT: 0
Case 3	AA present for 2 months (SALT: 2.0)	AP associated with UR7	Non‐surgical root canal treatment	4 months	Complete hair regrowth; SALT: 0

## 3. Case 1

A 20‐year‐old male patient was referred 3 years ago by the general medical practitioner due to discomfort in the lower quadrant. The patient reported dull, lingering discomfort in the lower right quadrant in addition to patchy hair loss of the scalp. With the patient′s consent, clinical photographs were taken of the alopecic bald patch on the patient′s scalp (Figure 1a,b). The patient was fit and healthy, with no known allergies. The clinical examination revealed a large restoration with dental caries at the mandibular right first molar (LR6).

**Figure 1:**
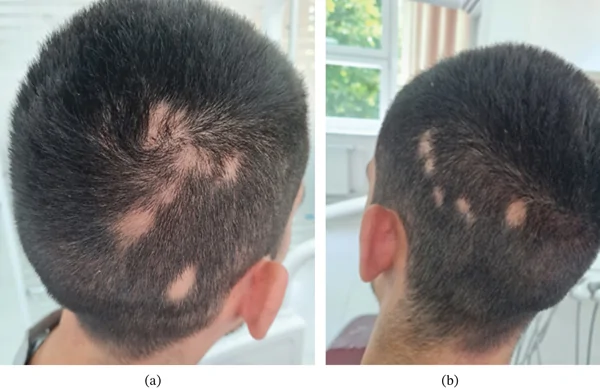
(a,b) Multiple alopecic patches noted on both (a) right and (b) left scalp region. A SALT (Severity of Alopecia Tool) score of 4.8 was provided by a dermatologist.

### 3.1. Clinical Findings

The LR6 was tender to vertical percussion and mildly tender to palpation in the buccal vestibule, suggesting apical inflammation. There was no swelling, no sinus tract and no mobility of the affected tooth. Periodontal assessment revealed no significant abnormalities. Six‐point pocket charting (6PPC) showed periodontal probing depths within normal limits (1–3 mm) at all sites at the LR6, with no bleeding on probing and attachment loss.

### 3.2. Radiographic Assessment

Periapical radiographs (Figure 2a,b) revealed suboptimal root canal treatment at the LR6. The mesiobuccal, mesiolingual and distal canals displayed short root fillings from the radiographic apex (3–7 mm). The obturation was undertapered and presented with voids, particularly in the coronal and middle thirds of the canals.

**Figure 2:**
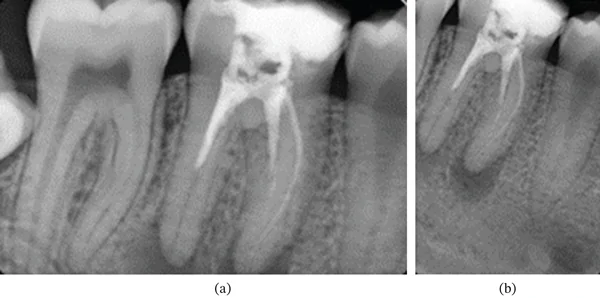
(a,b) Preoperative periapical radiographs of LR6 showing the apical lesions associated with both mesial and distal roots and evidence of existing root fillings, which were significantly short of the radiographic apex.

A well‐defined periapical radiolucency was evident around both the mesial and distal roots. No signs of root resorption, root fracture or overfilling were observed. The vertical and horizontal bone loss < 5*%*.

These findings, combined with clinical symptoms, supported a diagnosis of a previously treated tooth with symptomatic AP. Another deep carious lesion with poor prognosis was noted at the maxillary left first molar (UL6). Subsequently, treatment options were discussed with the patient either for re‐root canal treatment if the tooth was deemed restorable or extraction (Figure 2a, 2ba,b). However, the patient declined the proposed extraction, and the non‐surgical root canal retreatment was carried out following the verbal consent was obtained.

After the existing root fillings were removed, nonsetting calcium hydroxide paste was used in the root canal system as an intracanal medicament. After 2 weeks, the obturation was carried out using calcium silicate–based sealer (BioRoot‐RCS, Septodont, Saint Maur Des Fosses, France) and gutta‐percha using single‐cone obturation technique. Direct resin composite restoration (Tetric EvoCeram, Ivoclar, Liechtenstein) was placed at the LR6 as a coronal restoration.

At 1‐year follow‐up, periapical radiograph of the LR6 showed evidence of bone formation and reduction in size of the apical lesions (Figure 3). In addition, all alopecic areas at the 1‐year review showed evidence of new hair growth, except two separate patches on the right and left side of the head without new hair growth, which prompted the patient to decide for the extraction of UR6 (Figure 4a). Subsequently, at 18‐month review, the remaining alopecic patches were re‐filled with new hair, and all other areas exhibited further hair growth (Figure 4b).

**Figure 3:**
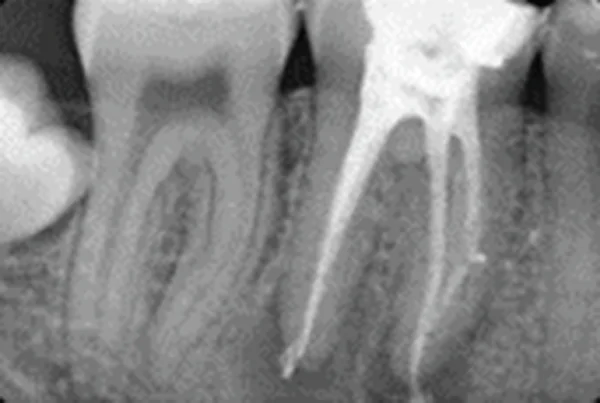
Periapical radiograph of the LR6 showing evidence of bone formation and reduction in size of the apical lesions at 1‐year review.

**Figure 4:**
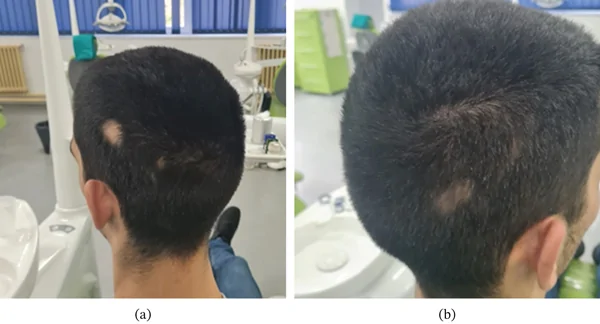
At (a) the 12‐month review, the alopecic patches had reduced, with a SALT score of 2.0, which further reduced to 1.8 at (b) the 18‐month review after the completion of the non‐surgical root canal retreatment at the LR6.

Periapical radiography showed the UL6 with extensive radiolucency extending to root canal system (Figure 5a). After obtaining the written consent from the patient, the UL6 was extracted (Figure 5b). In this respect, clinical findings at the 6‐month review after extraction of the UL6 showed the alopecic patch on the left side appeared to have been filled with new hair growth, with a SALT score of 0.4 (Figure 6a,b). Separately, periapical radiograph showed almost complete apical healing of the LR6 at 3‐year review (Figure 7) and all alopecic areas had been replaced with new healthy thick hair. Interestingly, there were no new episodes of alopecia recorded after 3 years (Figure 8a–c).

**Figure 5:**
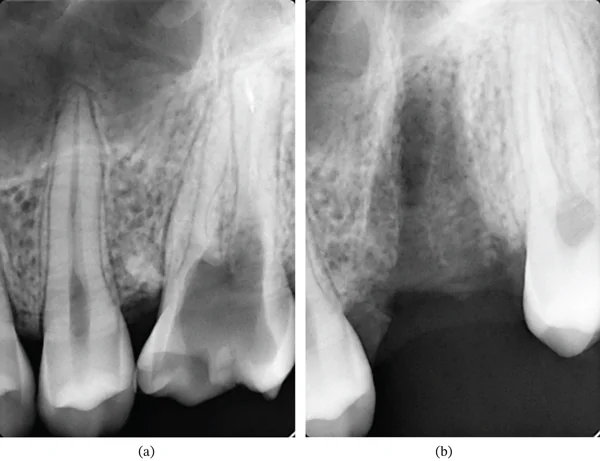
Periapical radiography of the UL6 with extensive radiolucency coronally extending to (a) root canal system and following the (b) extraction.

**Figure 6:**
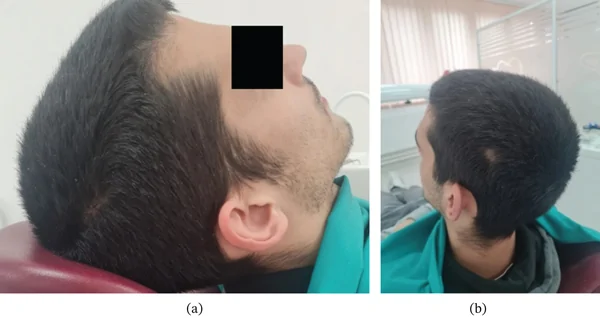
(a,b) Clinical findings at the 6‐month review after extraction of the UL6 showed the alopecic patch on the left side appeared to have been filled with new hair growth, with a SALT score of 0.4.

**Figure 7:**
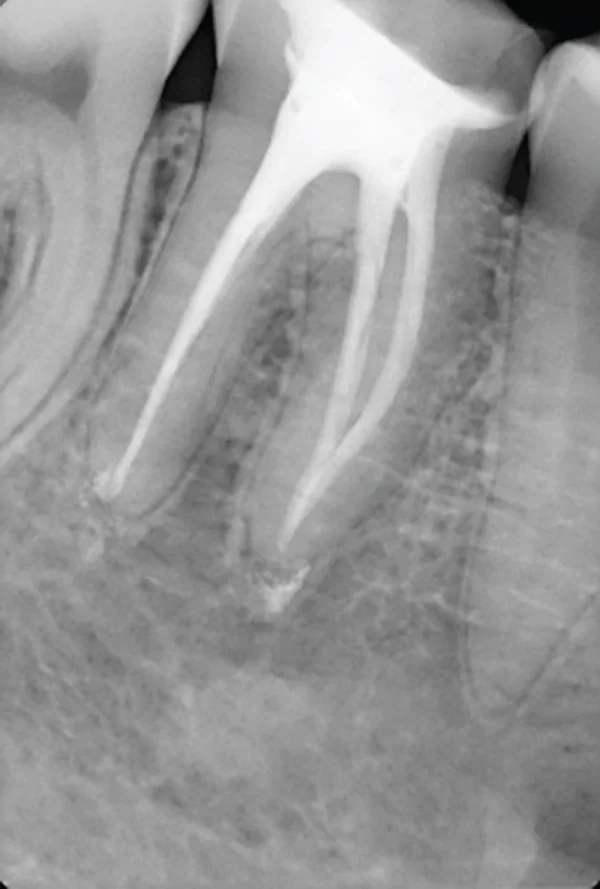
Periapical radiograph of LR6 at the 3‐year review showing almost complete bony healing and resolution of the periapical lesion.

**Figure 8:**
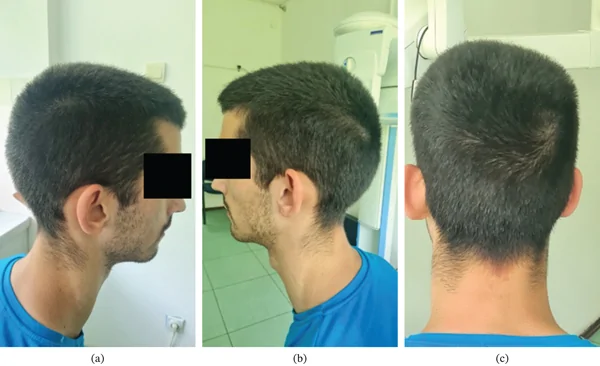
(a–c) Clinical findings at the 3‐year review showed no alopecic changes with a corresponding SALT score of 0.

## 4. Case 2

A 25‐year‐old male patient was seen at the Department of Endodontics at the Medical Faculty of Foca, University of East Sarajevo. The patient was fit and well, with no relevant medical history, allergies or medications noted. The patient was previously diagnosed with AA by the dermatologist with a SALT score of 4.0 and had been suffering with this condition for the past 2 years (Figure 9). The alopecic patch has become more obvious over the past year.

**Figure 9:**
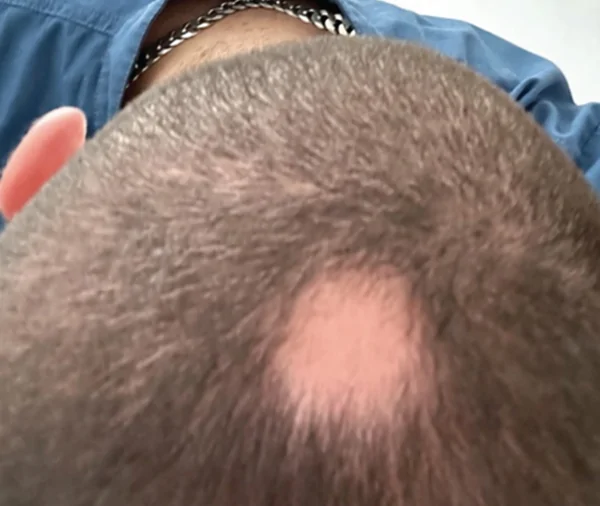
Photograph of patch of alopecia on patient′s scalp, with a SALT score of 4.0.

### 4.1. Clinical Findings

The patient reported sharp pain upon biting and chewing in the lower left quadrant. Clinical examination of the mandibular left first molar (LL6) revealed the tooth to be tender to vertical percussion, whereas palpation of the buccal mucosa elicited mild tenderness. The patient reported a previous episode of swelling a few weeks earlier, however there was no swelling or sinus tract and mobility at the time of clinical assessment. The periodontal pocket probing depths were between 1–3 mm, with no bleeding on probing and no clinical attachment loss.

### 4.2. Radiographic Assessment

Periapical radiographs showed an inadequately filled root canal system, especially in the distal canal, where the mesial root canals were short of the radiographic apex up to 3 mm. The obturation also appeared under‐tapered, with voids present, particularly in the apical third (Figure 10).

**Figure 10:**
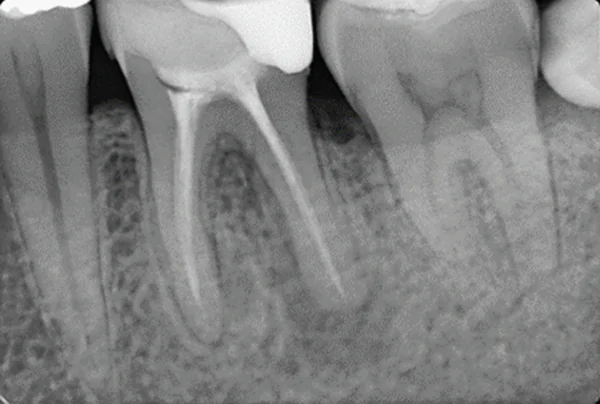
Preoperative periapical radiograph of the left mandibular first molar (LL6), which had short root fillings and periapical lesions associated with the distal root. The tooth was diagnosed as previously treated with symptomatic apical periodontitis.

A well‐circumscribed periapical radiolucency was present at the apex of the distal root, indicating chronic AP. The mesial root displayed widening of the periodontal ligament space, though no clear radiolucency was seen (Figure 10). The lamina dura was disrupted apically. No evidence of resorption, fractures or restorative overhangs was found. These findings were consistent with a diagnosis of symptomatic AP in a previously treated tooth (LL6). Following the patient′s written consent, clinical photographs were taken of the alopecic bald patch on the patient′s scalp (Figures 9). Following a discussion related to treatment options, the patient rejected a second attempt of root canal retreatment and opted for tooth extraction. Subsequently, the tooth was extracted (Figure 11). The alopecic zone exhibited hair growth after three, six and 9 months postextraction (Figures 12, 13, and 14a,b).

**Figure 11:**
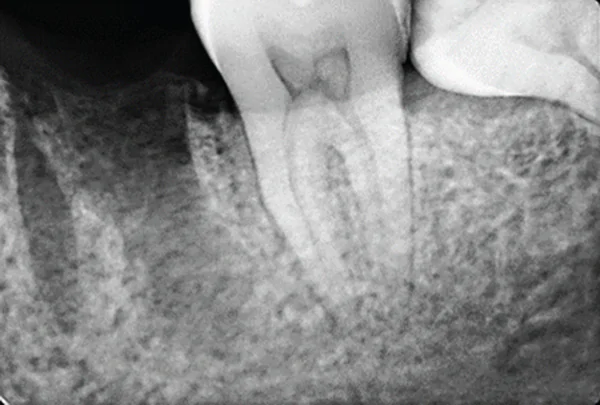
Postoperative periapical radiograph taken after extraction of the LL6.

**Figure 12:**
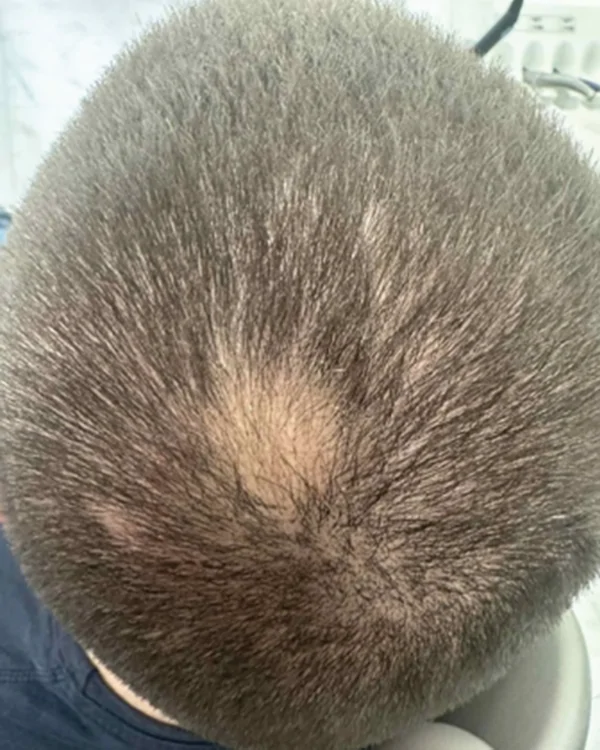
Hair growth noted at 3 months postextraction of LL6, with a SALT score of 2.0.

**Figure 13:**
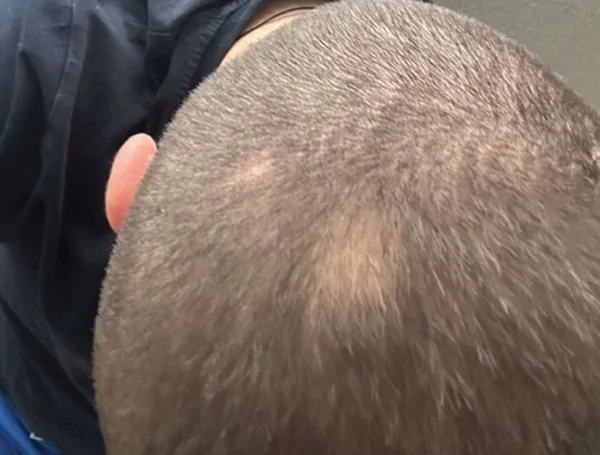
Further hair growth noted 6 months after the extraction of LL6 with a SALT score of 2.3.

**Figure 14:**
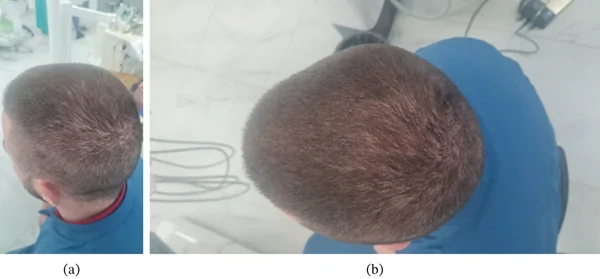
(a,b) Complete hair recovery at 9 months after the extraction of LL6 with a SALT score of 0.

## 5. Case 3

A 23‐year‐old male patient was seen at the Health Centre Zagreb, Croatia. The patient was fit and well, with no known allergies and medications; however, noticeable anxiety due to hair loss was noted. The patient was previously diagnosed with AA by the dermatologist with a SALT score of 2 and he has been suffering with this condition for the past 2 months (Figure 15).

**Figure 15:**
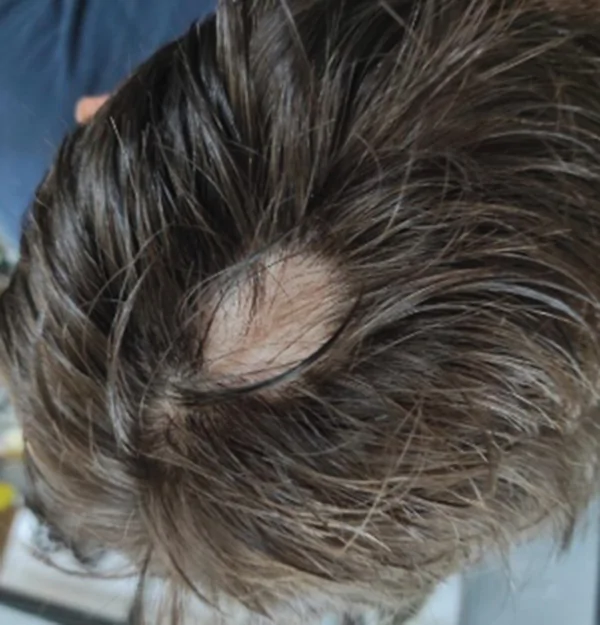
Photograph of patch of alopecia on patient′s scalp, with a SALT score of 2.

### 5.1. Clinical Findings

The patient reported a history of acute pain and facial swelling in the upper right quadrant approximately 9 months prior to examination, which was resolved spontaneously without treatment. At the time of presentation, the patient described occasional pressure sensitivity; however, there was no report of current pain on biting or swelling.

Clinical examination of the maxillary right second molar (UR7) revealed a negative response to cold testing using Endo‐Frost (−50°), tenderness to vertical percussion, mild tenderness to buccal palpation with no obvious mobility, sinus tract or swelling, the periodontal pocket depths were between 1 and 3 mm, with no bleeding on probing or attachment loss at the UR7.

### 5.2. Radiographic Assessment

Radiographic report indicated no evidence of previous endodontic treatment. The mesiobuccal, distobuccal and palatal canals appeared unenlarged and unfilled, with clearly visible pulp spaces. There was large, well‐circumscribed periapical radiolucency surrounded all three root apices, indicating chronic periapical bone loss. The periodontal ligament space was widened apically, and the lamina dura was disrupted at all root apices. No signs of internal or external root resorption, root fracture or apical radiopaque structures were identified (Figure 16).

**Figure 16:**
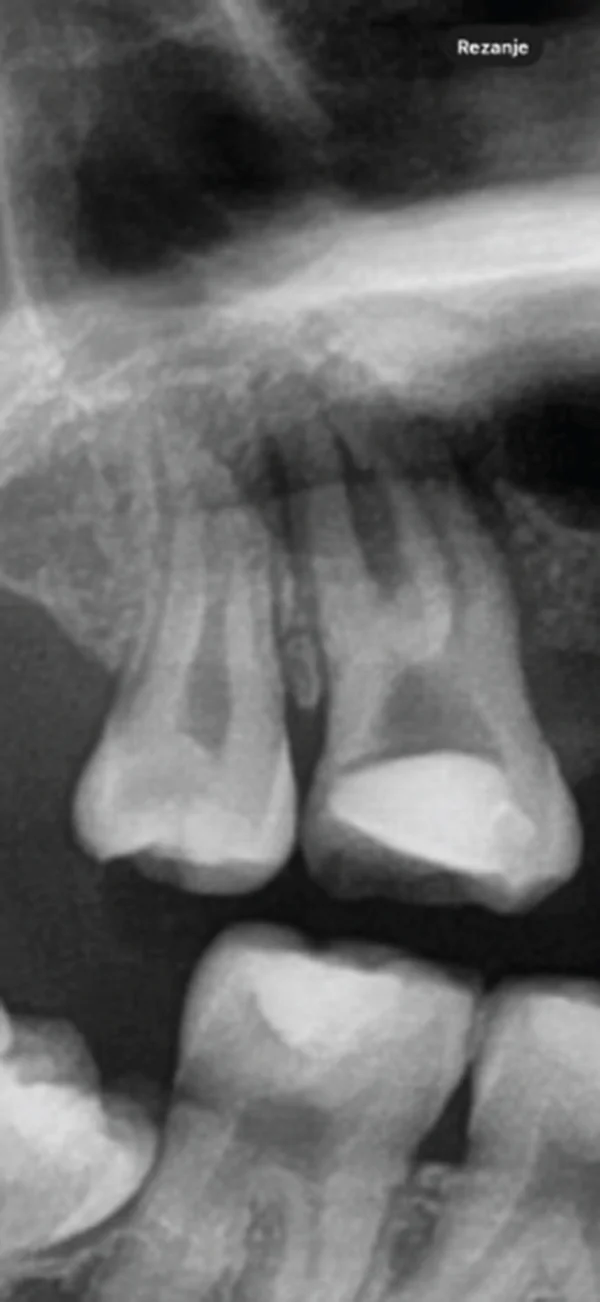
UR6 showed a large, well‐circumscribed periapical radiolucency associated with all three root apices, indicating apical periodontitis.

These clinical and radiographic findings supported a diagnosis of pulp necrosis with chronic AP, following a previously resolved episode of acute apical abscess.

With the patient′s consent, clinical photographs were taken of the alopecic bald patch on the patient’s scalp (Figure 15). Following a discussion of treatment options, the patient consented to endodontic treatment of UR7. Subsequently, non‐surgical root canal treatment was completed within one visit. The obturation was carried out using calcium silicate‐based sealer, BioRoot RCS (Septodont, Saint Maur Des Fosses, France) and sealed with glass ionomer cement (Fuji IX, GC Corporation, Tokyo, Japan) as provisional restoration. After 24 h, direct resin composite restoration (Tetric EvoCeram, Ivoclar, Liechtenstein) was placed at the UR7 as a coronal restoration. At the 4‐month review, periapical radiograph showed evidence of bone formation and reduction in size of the apical lesions at the UR7 (Figure 17). In addition, the alopecic zone exhibited complete hair growth after 4 months (Figure 18).

**Figure 17:**
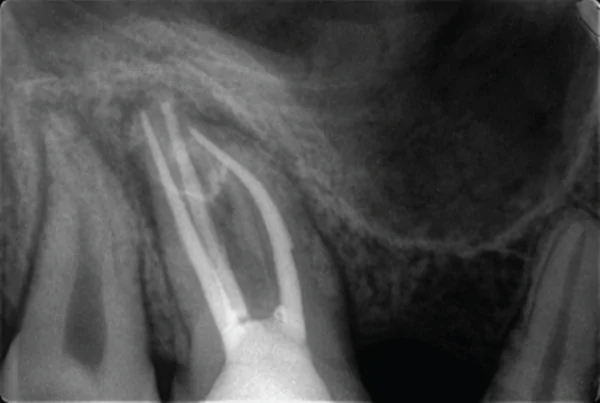
Periapical radiograph of UR6 at 4‐month review showing evidence of bony healing and resolution of the periapical lesion.

**Figure 18:**
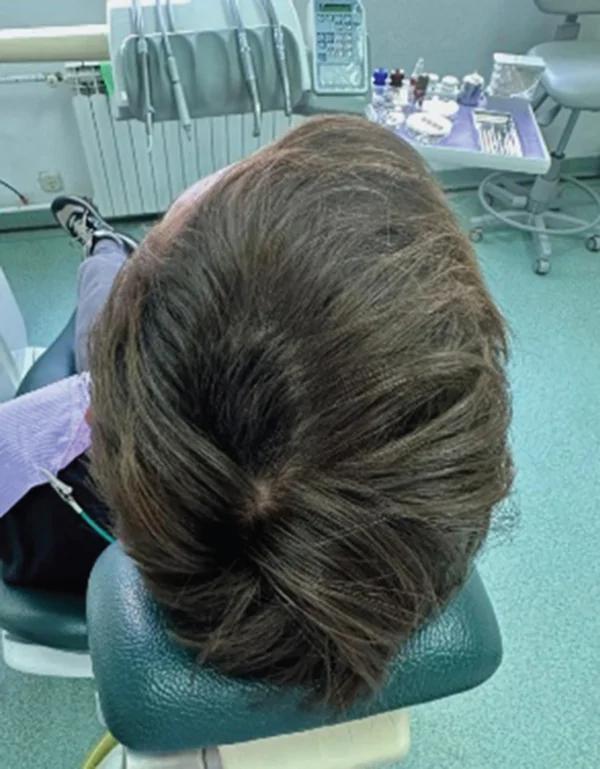
Complete hair recovery 4 months after the treatment with a SALT score of 0.

## 6. Discussion

There is a paucity of evidence on the possible association of AA and AP. Further evidence is required to clarify the exact mechanisms underlying these pathologies. The cellular and humoral immunity with both innate and adaptive responses orchestrate the inflammatory reaction, which might link these two conditions.

Endogenic immunoinflammatory agents, including cytokines, kinins, complement fragments, neuropeptides, lysosomal enzymes and fibrinolytic peptides, emerge due to the complex interactions between the host protector cells and oral irritants (mechanical, chemical or microbiological). These interactions divide immune responses into two categories: cell‐mediated and antigen antibody–mediated. Reported that the presence of immune complexes that are extensively distributed throughout the systemic circulation (particularly in acute oral infections), and there could be a plausible association between oral infections and AA [24].

The complexity of complex gram‐negative anaerobic microorganisms in apical lesions can be the reason for a general immune‐inflammatory response. These pathogens and their products, such as lipopolysaccharide (LPSs), could penetrate through the blood stream. In this respect, the microbial load and immune‐inflammatory host response might explain the mechanisms for the interactions between AP and AA [25].

Interestingly, a trigeminal‐sympathetic reflex mechanism offers an alternative explanation for the oral connection to AA. A centripetal response involving a triple‐neuron system is triggered by a distant mechanical or infectious stimulus, whereas centrifugal conduction involving the sympathetic nucleus near the terminal branches of the trigeminal nerve may cause vasoconstriction of the pilosebaceous follicle, causing several changes that lead to hair loss.

AP is generally considered to be driven by persistent infection of the root canal system, where necrotic pulp tissue and microbial biofilms (e.g., *Porphyromonas gingivalis* and *Fusobacterium nucleatum*) sustain chronic periapical inflammation. The bacterial products released act as PAMPs that stimulate toll‐like receptors (TLR2/4), NOD‐like receptors and the NLRP3 inflammasome. This results in prolonged release of IL‐1*β*, TNF‐*α*, IL‐6 and IFN‐*γ*, maintaining a Th1/Th17–biassed environment. In AA, the immune attack on the hair follicle is likewise sustained by IFN‐*γ* and JAK‐STAT signalling. Therefore, a long‐standing AP lesion may act as a systemic inflammatory trigger that lowers the threshold for AA onset in susceptible individuals.

Chronic AP generates continuous antigen presentation and broad T‐cell activation within apical tissues. Over time, this ‘immune noise’ can lead to bystander activation of autoreactive T cells that would otherwise remain dormant. At the same time, persistent infection and inflammation are known to impair Treg function. In AA, reduced Treg numbers and activity are central to the breakdown of tolerance at the hair follicle. AP could theoretically amplify this defect, possibly tipping the balance away from tolerance and towards autoreactivity against follicular antigens.

The microbial proteome of endodontic pathogens contains a wide range of antigens, some of which may resemble human peptides. Cross‐reactivity between bacterial antigens and follicular proteins, particularly melanogenesis‐associated antigens targeted in AA, could allow T cells primed during AP to recognize and attack hair follicle structures. However, this has not been demonstrated directly. This molecular mimicry model is supported by parallels in other infection‐linked autoimmune diseases, such as rheumatic fever following streptococcal infection.

In addition, both AP and AA lesions display remarkably similar chemokine signatures. In periapical granulomas, CXCL9 and CXCL10 are highly upregulated under the influence of IFN‐*γ*, driving recruitment of cytotoxic T cells and activated macrophages. In AA, the peribulbar ‘swarm of bees’ is maintained by the CXCL9/10/11–CXCR3 axis, amplifying CD8^+^ NKG2D^+^ T‐cell infiltration around the follicle (Ito et al. 2013). This overlap may suggest that systemic release of these chemokines and their downstream immune activation in AP could potentially reinforce the pathogenic loops that drive AA [26].

AP is not confined to the periapical region; therefore, bacterial components, inflammatory mediators and activated immune cells could spread into the circulation. This systemic dissemination contributes to the established links between oral infection and systemic diseases such as diabetes, rheumatoid arthritis and Alzheimer′s disease. In the same way, AP could provide a background of systemic type‐1 inflammation that makes the collapse of hair follicle immune response. In genetically predisposed hosts carrying HLA or ULBP/NKG2D variants, this systemic immune activation could be the critical ‘second hit’ that might trigger AA.

The observations reported in the present case series should be interpreted in the context of increasing evidence demonstrating that chronic oral inflammatory diseases may influence systemic inflammatory status. Recent investigations have shown that treatment of oral inflammatory conditions can be associated with favourable changes in biomarkers linked to endothelial dysfunction and systemic immune activation [27, 28]. Furthermore, smoking and other environmental factors are recognised modulators of both oral inflammatory burden and host immune responses. Although biomarker measurements were not available in the present cases, it is biologically plausible that elimination of chronic endodontic infection may reduce systemic inflammatory signalling and modify immune pathways implicated in AA. Nevertheless, this hypothesis remains speculative, and future prospective studies incorporating cytokine profiling, immune‐cell analyses and longitudinal clinical assessments are required to clarify whether treatment of AP influences the immunological mechanisms associated with AA.

As mentioned, several limitations should be acknowledged. AA is characterised by spontaneous remission and recurrence, and therefore improvement observed following dental treatment may represent, wholly or in part, the natural history of the disease. Other unidentified confounding variables may also have contributed to the observed outcomes. Consequently, these findings should be interpreted as preliminary clinical observations rather than evidence of causation.

## 7. Conclusions

This case series describes three patients in whom AA improved following treatment of associated AP. Although these findings demonstrate a potential temporal association, they do not establish causality. Shared immunological pathways provide biological plausibility for a potential relationship between these conditions. Larger prospective cohort studies, mechanistic immunological investigations and epidemiological analyses are required to determine whether a true biological association exists and to elucidate the possible underlying pathways. This could facilitate a multi‐disciplinary holistic and tailored approach for patients with AA by ensuring optimal oral health.

## Author Contributions

A.Z., K.G. and I.B. contributed to patient management and manuscript preparation. A.Z., A.B., S.K., F.P. and H.B. contributed to the manuscript preparation and critical revision.

## Funding

No funding was received for this manuscript.

## Disclosure

A.Z., A.B., S.K., F.P. and H.B. approved the final manuscript.

## Ethics Statement

Ethical approval was waived according to institutional policy because this manuscript reports retrospective clinical observations obtained during routine patient care and does not involve experimental procedures. This case series was prepared in accordance with the CARE (CAse REport) Guidelines. A completed CARE checklist has been submitted as a supporting information.

## Consent

Written informed consent for publication of clinical information and accompanying images was obtained from all patients.

## Conflicts of Interest

The authors declare no conflicts of interest.

## Supporting information


**Supporting Information** Additional supporting information can be found online in the Supporting Information section. File S1: CARE checklist for case reports.

## Data Availability

The data supporting the findings of this study are available from the corresponding author upon reasonable request.
