# Supplementary material for: Case Reports of Alopecia Areata Associated With Apical Periodontitis
Source: Case Rep Dent. 2026 Sep 30;2026:9446964. doi: 10.1155/crid/9446964 (PMC13627859; doi:10.1155/crid/9446964)
Supplement: Supplementary file 1 [file CRID-2026-9446964-s001.docx]

**Supplementary Material**

**CARE Checklist**

**Manuscript Title:** *Case Reports of Alopecia Areata Associated with Apical Periodontitis*

| **CARE Item** | **Description** | **Reported in Manuscript** |
| --- | --- | --- |
| Title | The words "case report" or "case series" and phenomenon of interest identified | Title |
| Keywords | 2-5 key words describing the case | Keywords section |
| Abstract 1 | Introduction and importance of case(s) | Abstract |
| Abstract 2 | Main symptoms and clinical findings | Abstract |
| Abstract 3 | Main diagnoses, interventions and outcomes | Abstract |
| Abstract 4 | Conclusion and lessons learned | Abstract |
| Introduction | Background and rationale with references | Introduction |
| Patient Information | Demographic characteristics, chief complaint, history | Case 1, Case 2, Case 3 |
| Clinical Findings | Relevant physical examination findings | Clinical Findings subsections |
| Timeline | Historical and current information organized chronologically | Timeline Table |
| Diagnostic Assessment | Diagnostic procedures and reasoning | Radiographic Assessment subsections |
| Diagnostic Challenges | Any challenges encountered | Not applicable |
| Therapeutic Intervention | Description of treatment procedures | Case Presentation |
| Follow-up and Outcomes | Clinical course and results | Follow-up sections and figures |
| Patient Perspective | Patient-reported perspective | Not reported |
| Informed Consent | Written informed consent obtained | Consent Statement |
| Discussion 1 | Strengths and limitations | Discussion and Limitations subsection |
| Discussion 2 | Relevant literature review | Discussion |
| Discussion 3 | Scientific rationale and interpretation | Discussion |
| Discussion 4 | Main lessons learned | Conclusion |
